# A systematic review of existing brief interventions for youth following suicide attempt: informing the development of an autism-adapted intervention

**DOI:** 10.3389/fpsyt.2026.1736168

**Published:** 2026-06-12

**Authors:** Stephanie J. Howe, Stephanie Andreasen, Jessica M. Schwartzman, Deinera Exner-Cortens, Sandy Thompson-Hodgetts, Jonathan A. Weiss, Kathleen Chaput, Meredith R. Maroney, Alexandra X. Jacobs, Jordan Mattingly, Carly A. McMorris

**Affiliations:** 1 Werklund School of Education, University of Calgary, Calgary, AB, Canada; 2 Alberta Children’s Hospital Research Institute, University of Calgary, Calgary, AB, Canada; 3 Department of Pediatrics, Keck School of Medicine, University of Southern California, Los Angeles, CA, United States; 4 Division of Developmental-Behavioural Pediatrics, Children’s Hospital Los Angeles, Los Angeles, CA, United States; 5 Department of Psychology, University of Calgary, Calgary, AB, Canada; 6 Department of Occupational Therapy, University of Alberta, Edmonton, AB, Canada; 7 Department of Psychology, York University, Toronto, ON, Canada; 8 Community Health Sciences, Cumming School of Medicine, University of Calgary, Calgary, AB, Canada; 9 Department of Counseling, School Psychology, and Sport, University of Massachusetts Boston, Boston, MA, United States

**Keywords:** autism, brief interventions, mental health services, safety planning, suicide prevention

## Abstract

**Background:**

Autistic youth are at elevated suicide risk, yet they face multiple barriers to mental health care, including a lack of interventions adapted to their unique needs and lived experiences. In the general population, brief interventions have been shown to reduce suicide risk during the high-risk period immediately following discharge from acute care for a suicide attempt. However, no suicide prevention intervention has been designed specifically for Autistic youth during this critical post-discharge period. The overarching aim of the current systematic review was to lay the groundwork for the development of a brief suicide intervention for Autistic youth following discharge from acute care. This review aimed to: (1) identify existing brief post-discharge interventions for general population youth (15–24 years) who have made a suicide attempt; (2) describe core strategies used; and (3) summarize evidence of intervention effectiveness.

**Methods:**

Following PRISMA reporting guidelines, five bibliographic databases were searched. Articles were included based on a rigorous selection process, and their quality was assessed using the Mixed Methods Appraisal Tool.

**Results:**

Twenty-four studies were included in the review, representing sixteen interventions. No studies reported including Autistic individuals, and Autistic people were explicitly excluded from two studies. Articles presented mixed findings, with eight studies indicating that the brief intervention was associated with a reduction in suicide outcomes. Common strategies included follow-up contact, safety planning, and teaching coping skills.

**Discussion:**

Several brief suicide interventions and strategies show promise for non-Autistic youth post-discharge, their relevance for Autistic youth remains unclear, underscoring the need for autism-adapted approaches informed by Autistic youth.

## 1 Introduction

Suicidality (i.e., suicidal ideation, suicide plans, attempts, and death by suicide; 1) is exceptionally common and often overlooked among individuals on the autism spectrum (hereafter referred to as ‘autism’). Autistic^1^ youth and adults are at 3.5 times greater risk of suicide attempts and deaths than the general population (5). A recent systematic review and meta-analysis (6) of 36 studies representing 48, 692 Autistic and possibly Autistic participants reported sizeable pooled prevalence estimates of suicidal ideation (34%), suicide plans (22%), and suicide attempts and behaviours (24%). These rates are considerably higher than those in the general population, which are estimated to be approximately 9% for suicidal ideation and 2-3% for suicide plans and attempts (7).

Although suicidality is a leading cause of death among youth in general (8), Autistic youth appear to face an even greater compounded risk, reflecting the intersection of developmental vulnerability and autism-related factors. Autistic people exhibit risk factors for suicidality related to autistic traits (e.g., impulsivity, emotion dysregulation, autistic burnout; 9–11), increased vulnerability to general risk factors such as co-occurring mental health conditions, victimization, loneliness, and adverse childhood experiences (12–15), as well as systemic risk factors including ableism, marginalization, and a lack of appropriate mental health care (16–18).

Given their heightened vulnerability to experiencing suicidality, Autistic youth are more likely than their non-Autistic peers to require acute mental health care, including psychiatric hospitalization and emergency department (ED) visits, and tend to remain in these systems longer (19–21). Despite these elevated rates of ED utilization, clinicians in these settings report feeling much less confident in assessing and addressing suicidality among Autistic individuals compared to their non-Autistic counterparts (22), primarily due to a lack of autism-specific or adapted risk assessment tools and suicide prevention approaches (23–25). Barriers to accessing mental health care also include a lack of clinician knowledge about autism, an unwillingness to tailor approaches to support the needs of Autistic individuals, systemic barriers (e.g., long wait lists), and client-based barriers (e.g., communication difficulties, finding accessing services too overwhelming; 16).

Although evidence-based suicide prevention interventions exist, they were developed for and validated in non-Autistic populations, and their effectiveness for Autistic people is currently unknown (11). The Safety Planning Intervention (SPI; 26) has been identified as a potentially effective approach for Autistic people due to its concrete stepwise structure (27). Recently, Goodwin and colleagues (28) developed the Autism Adapted Safety Plan (AASAP) in partnership with Autistic adults, incorporating changes such as simplified language and concrete examples. Initial findings support feasibility and acceptability of the AASAP among Autistic adults (29), but its clinical effectiveness and applicability to youth remain undocumented.

In the general population, prior suicidal behaviour, history of non-suicidal self-injury (NSSI), and previous suicide attempts are the strongest predictors of future suicide attempts and death by suicide (30). The risk of repeat suicide attempts is highest in the period immediately following a suicide attempt, with about 26% of repeat attempts occurring within the first month post-discharge, 41% within three months, and 73% within a year (31). While the prevalence of repeat suicide attempts among Autistic individuals remains unknown, evidence shows high rates of hospitalization and rehospitalization for mental health reasons (32, 33). For example, using a nationally representative United States database, Rast and colleagues (33) found that 36% of hospital admissions for Autistic adults in 2019 were due to mental health concerns, and 17% of these individuals were readmitted to the hospital within 30 days. Additionally, Schwartzman et al. (34) reported that Autistic youth and adults had an average of 2.92 suicide attempts, with some individuals reporting as many as 26 attempts. For Autistic youth, the post-discharge period may pose unique risks due to difficulties with transitions and disruptions to routine, heightened sensory and social demands of outpatient care, and challenges with communicating distress or suicidal intent (29, 35).

Effective intervention during the high-risk period of the weeks to months post-suicide attempt is critical (36). Several psychosocial interventions, including cognitive behaviour therapy (CBT), attachment-based family therapy, dialectical behaviour therapy (DBT), and interpersonal psychotherapy (IPT), have been applied to non-Autistic suicidal youth (36, 37). However, meta-analytic evidence suggests small and inconsistent efficacy of these interventions (38, 39). Even among participants that seek professional mental health services, their treatment engagement tends to be low (36, 40). While a typical course of treatment lasts 10–16 sessions for CBT (41), and 6–12 months for DBT (42), adolescents attend a median of only four outpatient sessions after a suicide attempt (36, 40, 43). Harpaz-Rotem and colleagues (40) found that children fully covered by medical insurance remained in mental health treatment for less than three of a recommended six months, attending less than one visit per month. Increased follow-up attendance may be related to higher illness severity, longer hospital stays, more sessions of therapy during hospitalization, and treatment by a mental health specialist (vs. a nonspecialist; 40, 43). Engagement challenges may be particularly pronounced for Autistic individuals due to barriers such as sensory overload in clinical settings, difficulty engaging with therapies that rely heavily on introspection or abstract cognitive strategies, and limited access to autism-informed clinicians (16, 44, 45). Taken together, full-length interventions are often not completed in full, and youth at high risk of suicide are not receiving the intended intervention dosage. Additionally, many traditional interventions are designed to be long-term (e.g., six months or more) and highly resource-intensive, potentially limiting the number of patients that may receive treatment (36). Given these limitations, there is a growing need to explore approaches that are less time-intensive but still effective.

Brief interventions, designed to quickly intervene during a period of elevated risk, may have the highest potential to improve access to care and effectively prevent repeat suicide attempts (36). Studies in healthcare generally report brief interventions as lasting about one to three sessions (46). These interventions typically focus on therapeutic engagement, safety planning, psychoeducation, affect regulation strategies, and follow-up contact (46, 47). By targeting the acute post-crisis period and focusing on practical, immediately applicable strategies, brief interventions have the potential to improve uptake and effectively prevent repeat suicide attempts. Brief interventions may also be a strong fit with Autistic cognitive and communication styles due to the frequent use of structured, goal-oriented formats and concrete, skills-based strategies, which align with recommendations for adapting mental health interventions for Autistic individuals (e.g., 27, 28, 48, 49, 98).

Importantly, interventions tailored to the needs and experiences of Autistic youth, whose developmental trajectories, communication styles, and support needs may differ substantially from those of non-Autistic youth (16, 50), are critically needed to increase the accessibility of essential mental health care and prevent premature mortality in this vulnerable population. Prior work, such as the AASAP (28), suggests general interventions can be successfully adapted for Autistic individuals. Thus, an essential first step in developing a tailored intervention is to identify existing interventions that are brief, evidence-based, and have the potential to be adapted for Autistic youth.

The current systematic review lays the groundwork for the development of a brief intervention *for* and *with* Autistic youth. Specifically, here we (1) identified existing brief interventions for youth post-suicide attempt; (2) described core strategies used, with attention to features relevant to potential adaptations for Autistic youth; and (3) summarized evidence of effectiveness.

Importantly, the current review is intended as a foundational step in the development of an autism-adapted brief post-suicide attempt intervention, rather than as a review of existing interventions specifically for Autistic youth. Despite consistently elevated rates of suicidality among Autistic individuals, there are currently no evidence-based suicide prevention interventions that have been developed or validated specifically for this population, including in the acute post-discharge period (51–53). This gap persists despite clear calls from Autistic individuals, families, and advocates identifying the development of adapted, accessible mental health and suicide prevention supports as a high research and service priority (54, 55). Given the limited likelihood of identifying autism-specific brief interventions, this review focuses on systematically identifying brief, evidence-based post-discharge interventions evaluated in the general youth population with the goal of informing the development of an autism-adapted intervention. Findings from the present review are currently being used to inform an ongoing project that involves the co-creation of an autism-adapted brief intervention with Autistic youth, caregivers, and clinicians.

## 2 Methods

### 2.1 Advisory board involvement

An advisory board was established to provide lived experience and clinical perspectives throughout the broader program of research focused on suicide prevention for Autistic youth. The board consisted of four Autistic young adults, one caregiver of an Autistic youth, and one clinician that supports Autistic youth experiencing suicidality. Advisors were invited to review and provide feedback on the planned search strategy and terms via email, though no major changes were suggested. Advisors were also provided the opportunity to contribute to data extraction; however, due to availability, this stage was completed by the research team.

Following data synthesis, a summary of review findings was shared with the advisory board, and members were invited to provide feedback during a group meeting and via email. Input focused on interpretation and contextualization of the findings rather than study selection or data extraction. Specifically, advisors highlighted autism-specific considerations that informed the framing and interpretation of results including perceived limitations of traditional therapeutic approaches (e.g., cognitive behavioural therapy), challenges forming and maintaining therapeutic relationships with non-autism-informed clinicians, and differences in how suicidality is experienced and communicated by Autistic individuals.

The first author drafted the manuscript and advisory board members were invited to contribute to the editing and revision of this manuscript as co-authors. Feedback from advisory board members related to language, framing, and clarity was incorporated. Advisory board members are actively involved in a parallel, ongoing project focused on the co-design of an autism-adapted brief suicide intervention based on findings from this review.

### 2.2 Search strategy

All search and analytic methods were preregistered with PROSPERO (CRD42024608279). Search terms were developed with a research librarian and reviewed by the project’s advisory board (see **Table 1**). No autism-specific population terms were included in the search strategy. This decision was made intentionally, as the primary objective of the review was to identify brief post-attempt interventions currently used with youth in the general population in order to inform the development of an autism-adapted intervention. Given the limited availability of autism-specific suicide interventions, restricting the search to autism-specific terms would likely have yielded few or no eligible studies. Instead, a broad population approach was used to maximize the identification of potentially relevant interventions.

**Table 1:** Search terms.

Suicidality terms AND	suicid* OR self-injur*, OR self-harm*, OR mental health crisis OR intentional overdose
Age terms AND	juven* OR youth OR young person* OR young adul* OR teen* OR adolesc* OR child* OR young people OR pediatric OR paediatric OR student*
Intervention terms AND	Trial* OR intervention* OR prevent* OR safety plan* OR treat* OR program* OR randomized OR RCT OR therap* OR counselling
Brief terms AND	brief OR one-time OR transitionary OR bridge
Post-attempt terms	post-discharg* OR attempt OR unsuccessful

*denotes wildcard search term.

Searches were conducted in PsycINFO, MEDLINE, Embase, CINAHL, and PubMed. Publication dates were restricted to articles published in the past 20 years (January 2004 to search date: October 19, 2024) to ensure that interventions included were likely to be relevant and consistent with current clinical practices. Searches were restricted to English-language studies conducted with human participants and published in a peer-reviewed journal or dissertation. Manual searches of Google Scholar were conducted using the same search terms as the database searches, limited to peer-reviewed articles in English. Additionally, reference lists of included articles and relevant systematic reviews were screened to identify further studies not captured in the database searches.

### 2.3 Selection criteria

**Figure 1:**
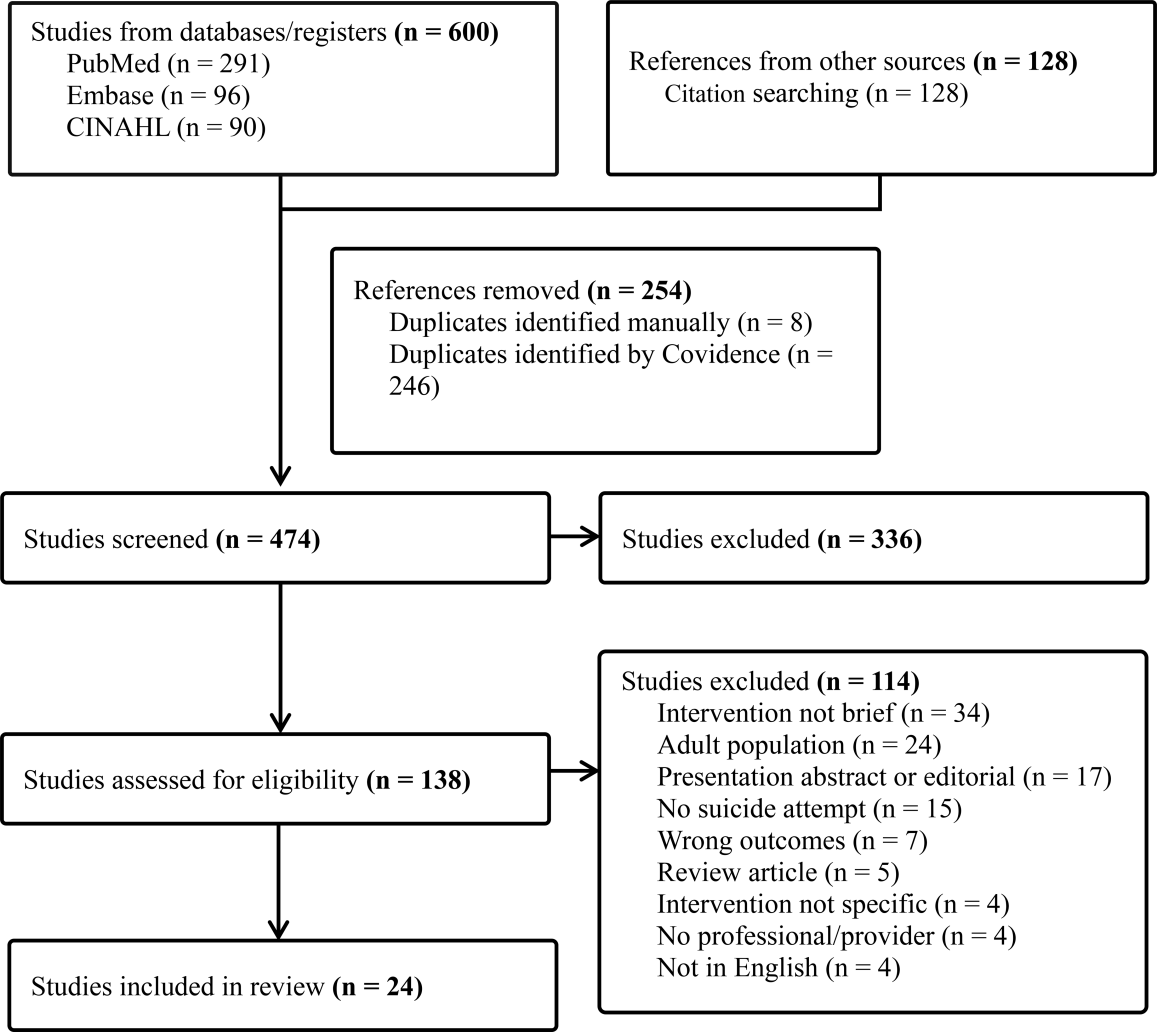
PRISMA diagram.

This review was conducted and reported according to the Preferred Reporting Items for Systematic Reviews and Meta-Analyses (PRISMA; 56) as seen in **Figure 1**. Covidence systematic review software (57) was used at all stages of the review. Two reviewers (S.J.H & S.A.) independently screened titles/abstracts and full texts. Discrepancies were resolved through discussion until consensus was reached.

Studies were eligible if they examined a brief psychosocial intervention (defined as up to four sessions or 240 minutes, excluding follow-up; 36) directly delivered to youth with a recent or lifetime suicide attempt. Articles were not restricted by study design, meaning that randomized controlled trials (RCTs), quasi-experimental (e.g., pre-post) designs, implementation research, and mixed methods or qualitative research were eligible to be included. Interventions had to involve contact with professionals or paraprofessionals (e.g., lay mental health workers, nursing assistants, volunteers). Parent and gatekeeper training, fully self-directed or automated interventions (e.g., mobile apps, postcards), and universal prevention programs (e.g., school-based education) were excluded to focus on interventions directly delivered to youth.

To ensure that the review findings would be relevant and appropriate for youth, the ages of participants in studies reviewed were restricted to ages 15 to 24 years, consistent with the United Nation’s definition of “youth” (UN, n.d.). When age ranges were not reported, studies were included if the mean age ±1 SD fell within this range (58). Because the review focused on interventions for youth post-suicide attempt, studies were eligible if ≥50% of participants had a history of suicide attempt, whether recent or lifetime. This criterion was adopted to accommodate for substantial variability in how suicidality and attempt history are operationalized and reported across the literature. When attempt history was not explicitly specified, studies were included if participants were recruited from high-risk clinical contexts (e.g., emergency departments [EDs] or inpatient units) where suicidal behaviour or imminent suicide risk are the primary reason for presentation.

### 2.4 Data extraction and quality assessment

Data pertaining to participants, study design, intervention characteristics, outcomes, and results were extracted into a standardized template on Covidence by two independent reviewers. Study quality was assessed using the Mixed Methods Appraisal Tool (MMAT; 59), which allows evaluation across multiple study designs. Intervention strategies were categorized by the first author, informed by study descriptions and clinical interpretation.

### 2.5 Identification of intervention strategies and components

Following data synthesis, an interpretive process consistent with recommendations for scoping reviews (60, 61) was undertaken to identify intervention components most relevant to be considered in the development of an autism-adapted brief intervention. This step was not intended as a formal analytic procedure, but rather as a theory- and community-informed synthesis to support intervention development. Core intervention strategies (e.g., safety planning, psychoeducation) were considered particularly relevant and summarized in the Results if they appeared across multiple included interventions with some evidence of effectiveness. Potential autism-specific considerations and adaptations to these strategies are included in the Discussion. These were informed by the authors’ familiarity with the autism and suicide literature, clinical experience, and consultation with both the research team and advisory board. Advisory board members provided feedback on the interpretation and contextualization of findings, including perceived fit of intervention strategies with their lived experiences. Strategies are presented as candidates for future adaptation and empirical evaluation rather than as validated recommendations.

## 3 Results

### 3.1 Study characteristics

Twenty-four studies were included, representing 16 unique interventions. Two sets of studies used overlapping samples (62–65) but were retained due to their distinct analyses and findings. Sample sizes ranged from 18 to 1,867 participants, representing a total of 7,904 participants across studies. No included studies reported having Autistic participants, and two studies (66, 67) explicitly excluded them.

Most studies were conducted in the United States (*n* = 14, 58%). Others took place in Australia (*n* = 2, 8%), Finland (*n* = 1, 4%), Israel (*n* = 1, 4%), Switzerland (*n* = 1, 4%), Iran (*n* = 1, 4%), France (*n* = 1, 4%), and India (*n* = 1, 4%). One international study (WHO SUPRE-MISS; 64, 65) used data from hospitals in five low- to middle-income countries (Brazil, India, Sri Lanka, Iran, and China), and two studies (68, 69) reported on regional subsets of this larger sample (Iran and India, respectively).

Nearly three quarters of studies (*n* = 17, 71%) were randomized controlled trials (RCTs). Other designs included prospective cohort (*n* = 2, 8%), retrospective cohort (*n* = 2, 8%), quasi-experimental (e.g., pre-post studies; *n* = 2, 8%), and historically controlled (*n* = 1, 4%). Although qualitative studies were eligible, none met selection criteria.

Most studies recruited youth presenting to emergency departments (EDs) or inpatient settings for suicidality, with some drawing on community referrals (e.g., 70, 71). While the review covered publications from 2004 onward, most were published in the last decade (*n* = 17, 71%), including five (21%) in 2024. Participants were primarily adolescents ages 13 to 16 years (*n* = 14, 58%), and nearly all studies (*n* = 23, 96%) had a higher number of girl participants than participants of other genders. Only two studies included a third category for gender (e.g., non-binary or gender non-conforming; 70, 72), and only one study specified the proportion of participants that identified as cisgender (66). Study characteristics and primary outcomes are reported in **Table 2**.

**Table 2:** Characteristics of studies included.

First author (Year)	Country	Study design	*N*	Overall *M* age ± *SD*	% Female	% lifetime SA	Groups (*n*)	Primary outcomes
Adrian (70)	USA	PCS	189	14.5 ± 2.0	62.4^a^	NR	CCC (189)	STB, SA, feasibility, acceptability, service use
Arvilommi (73)	Finland	RCT	161	32.1 ± 12.8	71	100	ASSIP (NR), CC (NR)	SA
Asarnow (62)	USA	RCT	181	14.7 ± 2.0	69	53	FISP (89), TAU (92)	SA, STB, service use
Bertolote (64)	Interna-tional (5 sites)	RCT	1867	Median = 23	58	100	BIC (922), TAU (945)	SA
Bookman (72)	USA	PCS	30	15.5 ± NR	46^b^	NR	CHATogether (30)	STB, feasibility, acceptability, depression, anxiety, conflict behaviour, health risk behaviour, help seeking attitudes
Haruvi Catalan (71)	Israel	RCT	26	13.4 ± 2.5	62	NR	WLC (NR), TAU (NR), IPT-A-SCI (NR)	STB, depression
Cwik (74)	USA	Pre-post	18	14.3 ± 2.2	92	100	New Hope (18)	STB, depression, service use
Czyz (75)	USA	RCT	36	15.4 ± 1.4	79	NR	MI-SafeCope (18), TAU (18)	STB, SA, feasibility, acceptability, satisfaction, motivation, self-efficacy
Czyz (76)	USA	RCT	80	15.2 ± 1.4	68	50	MI-SP + texts + booster call (18), MI-SP + texts (22), MI-SP + booster call (18), MI-SP (18)	STB, SA, feasibility, self-efficacy, coping behaviour
Fleischmann (65)	Interna-tional (5 sites)	RCT	1867	Median = 23	57	100	BIC (922), TAU (945)	SA
Goldstein (66)	USA	RCT	221	14.6 ± 1.6	81	67	ASAP Alone (61), ASAP + BRITE app (60)	STB, SA, NSSI, rehospitalization, satisfaction, usability
Gysin-Maillart (47)	Switzerland	RCT	120	37.8 ± 14.4	55	100	ASSIP (60), TAU (60)	STB, SA, depression, therapeutic alliance
Hassanzadeh (68)	Iran	RCT	632	23.9 ± 8.3 (BIC)	88 (BIC)	100	BIC (321), TAU (311)	SA
Hughes (63)	USA	RCT	181	14.7 ± 2.0	69	NR	FISP (89), control (92)	feasibility
Kennard (32)	USA	RCT	66	15.1 ± 1.5	89	80	ASAP (34), TAU (32)	STB, SA, NSSI, service use, satisfaction
77	Australia	Pre-post	141	31.5 ± 1.7	60	NR	AHBTC (141)	STB, rehospitalization, feasibility, acceptability
Messiah (78)	France	RCT	1040	NR^c^	66 (AlgoS 2+)	100	AlgoS 1 (248), AlgoS 2+ (232), TAU 1 (270), TAU 2+ (222)	SA
Rengasamy (48)	USA	RCT	142	15.0 ± 1.6	70	64	SCI (70), MCI (72)	STB, rehospitalization, acceptability
Schalley (79)	USA	RCS	612	NR^d^	68	NR	Caring Contacts (612)	Rehospitalization, feasibility, acceptability, fidelity, cost
Vijayakumar (80)	India	RCT	680	26.6 ± 9.9 (BIC)	51 (BIC)	100	BIC (320), TAU (360)	SA, completed suicide, depression
Wharff (21)	USA	HCT	250	15.6 ± 1.5 (FBCI)	76 (FBCI)	NR	FBCI (100), retrospective comparison (150)	Rehospitalization, feasibility
Wharff (81)	USA	RCT	139	15.5 ± 1.4	72	NR	FBCI (68), TAU (71)	STB, family empowerment, satisfaction
Wilhelm (82)	Australia	RCS	344	31.6 ± 11.3	75	NR	GCC (344)	Service use
Yen (83)	USA	RCT	50	15.7 ± 1.3	80	52	CLASP-A (25), ETAU (25)	SA, rehospitalization, treatment adherence, satisfaction

NR, not reported; RCT, randomized controlled trial; PCS, prospective cohort study; RCS, retrospective cohort study; HCT, historically controlled trial; WLC, waitlist control; TAU, treatment as usual; STB, suicidal thoughts and behaviours; SA, suicide attempts; NSSI, non-suicidal self-injury.

^a^ 5.4% nonbinary or genderqueer.

^b^ 13% gender non-conforming.

^c^ 35-50% of participants were reported to be between the ages of 18 and 35 years of age, depending on intervention group.

^d^ Participants were between 7 and 18 years of age.

### 3.2 Interventions identified

#### 3.2.1 Intervention characteristics

Sixteen brief interventions were included in the current review. Six (37%) included at least one session in the ED or an inpatient setting, while ten (63%) took place solely in an outpatient or community setting. Nearly half (*n* = 7, 44%) combined therapeutic sessions with follow-up contact, while six (37%) provided only therapeutic sessions, and three (19%) offered only follow-up contact post-discharge (e.g., postcards, letters, phone calls). Over half (*n* = 9, 56%) included a family component. Five (*n* = 5, 31%) could be completed in a single session. Common therapeutic approaches included cognitive behavioural therapy (CBT; *n* = 7, 44%), brief contact (BIC; *n* = 4, 25%), motivational interviewing (MI; *n* = 3, 19%), and narrative therapy (*n* = 2, 13%). Full intervention characteristics are shown in **Table 3**.

**Table 3:** Summary of intervention characteristics.

Intervention	Setting	Type	No. of sessions (Length)	Follow-up contact	Therapeutic approach	References
AHBTC	Community	Individual	NR (25–125 min.)	None	SFBT, CBT, DBT	Mansfield (77)
AlgoS Algorithm	Outpatient	Individual	None	Crisis card or phone call and 4 postcards	BCI	Messiah (78)
As Safe as Possible (ASAP) and BRITE App	Inpatient, Community	Individual	3-4 (~60 min.)	2 coaching phone calls	MI	Kennard (32) Goldstein (66)
Attempted Suicide Short Intervention (ASSIP)	Outpatient	Individual	3-4 (60–90 min.)	Letters over 2-year period	Humanistic, Narrative	Gysin-Maillart (47) Arvilommi (73)
Brief Intervention and Contact (BIC)	Inpatient (ED), Outpatient	Individual	1 (60 min.)	9 phone calls or visits	BCI	Bertolote (64) Fleischmann (65)Hassanzadeh (68) Vijayakumar (80)
Caring Contacts	Outpatient	Individual	None	6 personalized postcards	BCI	Schalley (79)
Behavioural Health Crisis Care Clinic (CCC)	Community	Individual, Family	4 (50–90 min.)	None	CAMS	Adrian (70)
CHATogether	Outpatient	Family	4-6 (60 min.)	None	CBT, Drama therapy	Bookman (72)
Coping Long Term with Active Suicide in Adolescents (CLASP-A)	Inpatient, Outpatient	Individual, Family	4 (30 min.)	11 phone calls	ACT. FITT	Yen (83)
Family-Based Crisis Intervention (FBCI)	Inpatient (ED)	Family	1 (60–90 min.)	None	CBT, Narrative	Wharff (21, 81)
Family Intervention for Suicide Prevention (FISP)	Inpatient (ED), Outpatient	Individual, Family	1 (NR)	3 phone calls	CBT	Asarnow (62) Hughes (63)
Green Card Clinic (GCC)	Outpatient	Individual	4 (NR)	None	CBT	Wilhelm (82)
Interpersonal psychotherapy for adolescents (IPT-A-SCI)	Outpatient	Individual, Family	5 (50 min.)	4 personalized emails	IPT	Haruvi Catalan (71)
MI-SafeCope/MI-SP	Inpatient	Family, Individual	1 (90 min.)	1 parent and 1 youth phone call	CBT, MI	Czyz (75, 76)
New Hope	Community	Family, Individual	1-2 (60–120 min.)	None	CBT, MI	Cwik (74)
Single Call Intervention (SCI) or Multiple Calls Intervention (MCI)	Outpatient	Individual, Family	None	1 (SCI) to 6 (MCI) phone calls	BCI	Rengasamy (48)

SFBT, Solution-Focused Brief Therapy; DBT, Dialectical Behaviour Therapy; BCI, Brief Contact Intervention; MI, Motivational Interviewing; CBT, Cognitive Behaviour Therapy; IPT, Interpersonal Psychotherapy; ACT, Acceptance and Commitment Therapy; FITT, Family Intervention Telephone Tracking; CAMS, Collaborative Assessment and Management of Suicidality.

#### 3.2.2 Intervention strategies

A summary of intervention strategies and relevant examples is provided in **Table 4**. The most common approaches are briefly described below in order of frequency.

**Table 4:** Summary of intervention strategies.

Strategy/content	Examples	Interventions
Identifying/reinforcing youth protective factors (*n* = 7, 44%)	Eliciting reasons for living, hope, social support, youth and family strengths, positive reinforcement, family connectedness	ASAP + BRITE; Caring Contacts; CCC; CLASP-A; FBCI; FISP; New Hope
Assessment (*n* = 7, 44%)	Assessing risk; taking psychosocial history; conducting case conceptualization (e.g., identifying target problem), functional/chain analysis (ASAP)	AlgoS; ASAP + BRITE; ASSIP; CHATogether; GCC; IPT-A-SCI; SCI/MCI
Creating a safety plan (*n* = 9, 56%)	Creating list of emergency/crisis support contacts, support people, coping strategies, calming activities and/or reasons for living	AHBTC; ASSIP; ASAP + BRITE; IPT-A-SCI; FBCI; FISP; MI-SafeCope/MI-SP; New Hope; SCI/MCI
Teaching/practicing coping skills and strategies (*n* = 9, 56%)	Practicing relaxation, self-soothing, and distraction; identifying thoughts, triggers, feelings, and behaviours; using tools (e.g., emotion thermometer); teaching emotion regulation, distress tolerance, behavioural activation, cognitive restructuring, and savoring and switching (ASAP)	ASAP + BRITE; BIC; Caring Contacts; CCC; FBCI; FISP; GCC; IPT-A-SCI; New Hope
Teaching/practicing other youth skills (*n* = 6, 38%)	Developing social, communication, interpersonal, conflict resolution, and problem-solving skills	ASAP + BRITE; CCC; CHATogether; CLASP-A; GCC; IPT-A-SCI
Parent training (*n* = 4, 25%)	Providing psychoeducation about how to manage crisis; clarifying role as coach; practicing communication and conflict resolution skills	ASAP + BRITE; CCC; CHATogether; MI-SafeCope/MI-SP
Follow-up (*n* = 10, 63%)	Providing reminders, positive affirmations, personalized emails/letters/postcards; conducting check-in phone calls; monitoring risk and treatment adherence; using app notifications	AlgoS; ASAP + BRITE; ASSIP; BIC; Caring Contacts; CLASP-A; IPT-A-SCI; MI-SafeCope/MI-SP; SCI/MCI
Lethal Means (*n* = 4, 25%)	Providing education; restricting access; using tools (e.g., lock boxes)	ASAP + BRITE; CCC; FISP; SCI/MCI
Connecting to follow-up treatment (*n* = 7, 44%)	Providing referrals, education/care navigation; facilitating transitions	AHBTC; BIC; CCC; FISP; IPT-A-SCI; New Hope; SCI/MCI
Psychoeducation about suicide (*n* = 8, 50%)	Labelling suicide as a serious problem; teaching epidemiology, risk and protective factors; providing psychoeducation about depression/mental health	ASAP + BRITE; BIC; CCC; FBCI; FISP; GCC; New Hope; SCI/MCI
Exploring and Processing (*n* = 6, 38%)	Processing context/events that led up to suicidal behaviour; focusing on the therapeutic alliance; understanding underlying problems; providing psychodynamic therapy, drama therapy/role playing, narrative therapy	ASSIP; CCC; CHATogether; FBCI; GCC; IPT-A-SCI
Enhancing motivation for behaviour change and adherence (*n* = 5, 31%)	Engaging in motivational interviewing, troubleshooting barriers, enhancing commitment to safety	ASAP + BRITE; FISP; GCC; MI-SafeCope/MI-SP; New Hope

##### 3.2.2.1 Follow-up contact

The most frequent strategy (*n* = 10, 63%) was follow-up contact, typically by phone (*n* = 7) or written communications in the form of letters, postcards, or emails (*n* = 4). The number of contacts ranged from one (51 [SCI]) to eleven (83 [CLASP-A]) and were typically made by the discharging clinician (e.g., psychologist, doctor, social worker, or nurse). Clinician phone calls often included review of safety plans, assessment of suicide risk, and the provision of additional support or referral if needed (e.g., 65 [BIC]). Written communications included positive affirmations, reminders about protective factors (e.g., identified reasons for living), coping strategies, and general mental health tips (e.g., 52 [Caring Contacts]).

##### 3.2.2.2 Safety planning

Over half of interventions (*n* = 9, 56%) incorporated safety planning. Czyz et al. (75) reported that their MI-SafeCope intervention was modelled after Stanley and Brown’s Safety Planning Intervention (SPI; 45). Two studies explicitly cited the SPI in their descriptions of safety planning (47 [ASSIP]; 71 [IPT-A-S]). The Single- or Multiple-Call Interventions (51) utilized a safety plan based on the National Suicide Prevention Lifeline’s framework. The remaining studies did not specify their approach and likely used generic or adapted versions. Safety plans were typically a document with coping strategies and reminders that could be easily referred to in times of crisis. For example, in the Attempted Suicide Short Intervention Program (ASSIP; 47), adolescents worked with a therapist to create a personal list of long-term goals, warning signs, and safety strategies, which was printed on a credit-card sized leaflet for the patient to take home. Safety plans could also be digital, as in the BRITE mobile application, which included a safety plan and emotion regulation skills personalized to the adolescent (66).

##### 3.2.2.3 Teaching coping skills

Nine interventions (56%) emphasized teaching and practicing coping skills (e.g., relaxation, distress tolerance, behavioural activation) with participants, as well as the identification of triggers, thoughts, feelings, and behaviours. As Safe as Possible (ASAP; 32, 66) contained three modules that relied heavily on coping skills delivered within an MI framework. Core skills taught to adolescents were “savouring” (recalling and visualizing positive experiences), affect switching (practicing flexibility to activate positive affect when in a ruminative cycle), and distress tolerance (using relaxation, self-soothing, and distraction to manage painful emotions; 32). Some interventions used tools to teach and reinforce concepts. For example, the Family Intervention for Suicide Prevention (FISP; 20, 62) used an “emotional thermometer” to identify feelings and physical, cognitive, and behavioural reactions to triggers as well as a “hope box”, which expanded on patients’ safety plans with concrete objects to cue the use of coping strategies.

##### 3.2.2.4 Providing psychoeducation

Half of the interventions (*n* = 8, 50%) included a focus on psychoeducation. For example, Brief Intervention and Contact (BIC; 64, 65, 68, 69) included a one-hour information session in the ED, in which patients received education about suicidal behaviour as a sign of distress, risk and protective factors, epidemiology of suicide, and alternatives to suicidal behaviour. The single session of FISP delivered in the ED (20, 62) focused on framing a suicide attempt as a problem requiring action, educating families about the importance of outpatient mental healthcare, and underscoring the importance of restricting access to lethal means. Finally, the New Hope intervention for Apache adolescents (74) included an educational video with Indigenous actors participating in dramatic vignettes and messages from Elders about the seriousness of suicide and its impact on the community.

##### 3.2.2.5 Identifying and reinforcing protective factors

Seven interventions (44%) identified and reinforced protective factors of the youth and family, such as strengths, reasons for living, and connectedness. FISP (62, 63) included a discussion of positive attributes and interactions of the youth and family, while Coping with Long-term Active Suicide Program for Adolescents (CLASP-A; 83) targeted four risk factors for suicide: hopelessness, family and social support, problem-solving skills, and treatment adherence.

##### 3.2.2.6 Assessment

Seven interventions (44%) incorporated assessment, which included assessment and continued monitoring of suicide risk, as well as appraisal of key problems and contributing factors. For example, follow-up phone calls in the Single- or Multiple-Call Interventions (SCI/MCI; 51) began with assessment of the adolescent’s suicide risk using the Columbia-Suicide Severity Rating Scale (C-SSRS) (84, 85) as well as asking youth to rate their confidence in using their safety plan. This information aided clinician’s determination of whether further support or referral was necessary. ASAP (66, 67) used a chain analysis (also known as functional analysis) to examine the function of a behaviour in-depth, including related vulnerabilities, cognitive and behavioural factors, and stressors. Additionally, Interpersonal Psychotherapy for Suicidal Children and Adolescents (IPT-A-SCI; 71) began with a review of the patient’s interpersonal relationships using an interpersonal inventory and conceptualizing interpersonal problem areas to work on in future sessions.

##### 3.2.2.7 Connecting to follow-up treatment

Seven interventions (44%) stressed the importance of connecting patients to follow-up treatment. For instance, the Allied Health Brief Therapies Clinic (AHBTC), as described by Mansfield and colleagues (77), delivered brief support to individuals in suicidal crisis, and assisted their transition to ongoing engagement with community-based, private, and primary care providers. Likewise, the Crisis Care Clinic (CCC; 70) offered three to five therapeutic sessions and a case manager that supported families in navigating the complexities of the mental health care system, coordinating and scheduling appointments, and otherwise reducing barriers to care.

### 3.3 Study findings

#### 3.3.1 Suicide attempts and deaths

Fourteen studies (58%) measured the impact of brief interventions on suicide attempts and/or deaths. Three RCTs (47, 65, 69) found that participants receiving the intervention were less likely to attempt suicide after the intervention than those in treatment as usual (TAU). For example, ASSIP was associated with an 80% reduced risk of repeat suicide attempts (47). In the WHO SUPRE-MISS trial, significantly fewer suicide deaths occurred in those that participated in BIC than those that completed TAU (65). Across the entire WHO-SUPRE-MISS sample, there were no main intervention effects on repeat suicide attempts; however, rates of re-attempts varied substantially across sites (64). Suicide attempts and deaths were lower in those that completed BIC than TAU in India (69), but no group differences were found in Iran (68). A pilot trial of CCC also reported a significant decrease in clinician-reported suicide attempts between baseline and post-CCC intervention (70).

Six RCTs did not find a significant difference in suicide attempts and/or deaths between intervention and TAU (62[FISP]; 64 [BIC]; 68 [BIC]; 75 [MI-SafeCope]; 67 [ASAP]; 83 [CLASP-A]).

Four studies compared interventions directly. Czyz and colleagues (76) compared a motivational interviewing enhanced safety plan (MI-SP) alone to MI-SP with added text messages and/or a booster call. These authors found that there were no significant differences in suicide attempts between conditions. Goldstein and colleagues (66) compared the ASAP intervention alone, the BRITE mobile application alone, ASAP and BRITE together, and TAU. Participants hospitalized for a suicide attempt and randomized to BRITE had a lower rate of subsequent attempts and a greater time to re-attempt. Another study (86) investigated the effectiveness of an algorithm (AlgoS) that assigned patients that had attempted suicide to a brief intervention based on the number of previous attempts. “First-attempters” (i.e., the attempt they presented for was their first lifetime suicide attempt) received a card with a crisis phone number at discharge, and “multi-attempters” received a follow-up phone call from a psychologist and, if necessary, an emergency consultation and/or a series of postcards. First-attempters in the AlgoS group were less likely to re-attempt suicide than first-attempters receiving TAU, while multi-attempters receiving AlgoS had similar rates of re-attempted suicide as their TAU counterparts (86). Finally, Arvilommi and colleagues (73) compared ASSIP with crisis counselling and did not find group differences in subsequent suicide attempts.

#### 3.3.2 Suicidal thoughts and behaviours

Thirteen studies (54%) assessed effects on suicidal thoughts and behaviours (i.e., preparatory acts, aborted or interrupted attempts; 84, 85). Three (70 [CCC]; 74 [New Hope]; 87 [AHBTC]) reported reductions, while five studies (62 [FISP]; 71 [ITP-A-SCI]; 75 [MI-SafeCope]; 47 [ASSIP]; 67 [ASAP]) found no effects.

While not directly measuring suicidal thoughts and behaviours, Bookman and colleagues’ (72) pilot study evaluating the effectiveness of the CHATogether family-centered intervention found trend improvements on the Concise Health Risk Tracking Self-Report (CHRT-SR9) on factors indicating suicide risk, including pessimism, helplessness, and despair. Wharff and colleagues (81) found that adolescents that participated in the Family-Based Crisis Intervention (FBCI) did not differ from adolescents that completed TAU on the Reasons for Living Inventory (RFL-A).

Three studies compared multiple interventions on their impact on suicidal thoughts and behaviours. MI-SP with added texts/booster calls showed no added benefit (76). Rengasamy and Sparks (51) found that adolescents assigned to a multiple-call intervention (MCI) reported significantly lower rates of suicidal behaviour than adolescents assigned to a single-call intervention (SCI; 51). Finally, there were no group differences in suicidal ideation found when the ASAP brief intervention was compared to ASAP with the addition of the BRITE mobile app (66).

#### 3.3.3 Hospital readmissions and service use

Six studies (*n* = 6; 25%) examined rehospitalization. Five found reduced likelihood that participants would return to the hospital for mental health concerns within the follow-up period (74 [New Hope]; 66 [ASAP]; 47 [ASSIP]; 87 [AHBTC]; 81 [FBCI]), while one (83 [CLASP-A) found no differences.

Four studies (17%) found brief interventions increased participants’ likelihood of being connected to, and continuing with, outpatient mental health care (70 [CCC]; 62 [FISP]; 74 [New Hope]; 68 [BIC]). For example, New Hope, an intervention developed for adolescents of the White Mountain Apache tribe who had recently attempted suicide, found that participants were significantly more likely to be engaged in outpatient mental health care at one- and two-months post-intervention compared to pre-intervention (74).

#### 3.3.4 Intervention impact on other mental health outcomes

Four studies (*n* = 4, 17%) assessed depressive symptoms. One study reported significant reductions in symptoms of depression post-intervention (74 [New Hope]), while three did not indicate significant effects (71 [IPT-A-SCI]; 47 [ASSIP]; 69 [BIC]). A small pilot study reported trend reductions in both depression and anxiety following participation in the CHATogether family intervention (72).

#### 3.3.5 Intervention feasibility and acceptability

Fourteen studies (*n* = 14; 58%) reported on their brief intervention’s feasibility and acceptability. All studies reported meeting acceptable benchmarks of feasibility and acceptability; however, studies varied significantly in their threshold for what constituted an acceptable outcome (see Study Quality).

### 3.4 Study quality

Study quality ratings using the Mixed Methods Appraisal Tool (MMAT) criteria are shown in **Table 5**. All studies passed the two screening questions confirming they were empirical and suitable for MMAT appraisal. However, many lacked key methodological details (e.g., randomization methods, blinding of outcome evaluators, completion rates), leading to frequent “can’t tell” ratings. Several also had high attrition rates, resulting in “no” ratings for complete outcome data. For example, Gysin-Maillart et al. (47) and Yen et al. (83) lost over 35% of participants in one or more groups to follow-up, significantly increasing risk of bias. Other studies had poor adherence or incomplete intervention delivery (e.g., >30% did not complete the intervention). For example, in Rengasamy and Sparks’ (48) comparison of two phone interventions (SCI/MCI), phone contact was made with only 39% of SCI and 32% of MCI participants.

**Table 5:** Quality ratings of studies included as measured by the MMAT.

Quantitative randomized controlled trials					
Study	Is randomization appropriately performed?	Are the groups comparable at baseline?	Are there complete outcome data?	Are outcome assessors blinded?	Did participants adhere to the intervention?
Arvilommi (73)	Y	CT	N	Y	Y
Asarnow (62)	Y	N	Y	Y	CT
Bertolote (64)	Y	Y	Y	Y	Y
Haruvi Catalan (71)	CT	CT	CT	CT	Y
Czyz (75)	Y	Y	Y	CT	Y
Czyz (76)	CT	CT	CT	CT	Y
Fleischmann (65)	Y	Y	Y	Y	Y
Goldstein (66)	Y	Y	Y	Y	Y
Gysin-Maillart (47)	Y	Y	N	CT	Y
Hassanzadeh (68)	Y	N	Y	Y	CT
Hughes (63)	CT	Y	N	CT	Y
Kennard (32)	Y	Y	Y	Y	Y
Messiah (78)	Y	Y	Y	Y	Y
Rengasamy (48)	Y	Y	Y	CT	N
Vijayakumar (80)	Y	Y	Y	N	Y
Wharff (81)	CT	Y	N	Y	Y
Yen (83)	Y	Y	N	Y	N
Quantitative non-randomized					
Study	Are participants representative of the population?	Are measurements appropriate?	Are there complete outcome data?	Are confounders accounted for?	Is intervention administered as intended?
Adrian (70)	Y	Y	N	Y	Y
Quantitative descriptive					
Study	Is the sampling strategy relevant?	Is the sample representative of the population?	Are the measurements appropriate?	Is the risk of nonresponse bias low?	Is the statistical analysis appropriate?
Schalley (79)	Y	Y	N	Y	Y
Cwik (74)	Y	Y	Y	N	Y
Mansfield (77)	Y	Y	N	N	Y
Wharff (21)	Y	Y	Y	Y	Y
Wilhelm (82)	Y	Y	Y	N	N
Mixed methods					
Study	Is there rationale for mixed methods?	Are components effectively integrated?	Are the outputs adequately interpreted?	Are divergences/inconsistencies addressed?	Do components adhere to quality criteria?
Bookman (72)	Y	Y	N	Y	N

Y, yes; N, no; CT, can’t tell.

## 4 Discussion

Autistic youth are at heightened risk for experiencing suicidality, yet little is known about which interventions may effectively prevent suicide in this population. As a first step in adapting a brief intervention for Autistic youth, this systematic review identified 24 studies representing 16 unique interventions that had been used with youth in the general population.

### 4.1 Aim 1: identifying brief interventions for youth who have attempted suicide

All interventions targeted youth in the general population, with one exception (74), which was tailored to an Indigenous community. No studies specified whether participants had neurodevelopmental conditions, and two explicitly excluded Autistic youth. This pattern reflects a broader historical trend of excluding Autistic individuals from mainstream mental health research and clinical trials, which has contributed to disparities in service provision and outcomes (18, 88). Drivers of this exclusion include provider uncertainty, stigma, communication and cognitive differences, and perceived diagnostic complexity (18, 88). As a result, there are limited validated suicide assessment tools (e.g., 89), few evidenced-based intervention options, and heightened barriers to care for Autistic youth (16), reinforcing systemic inequities. While recent progress has been made in developing suicide assessment and intervention tools for Autistic adults (e.g., 28, 90, 91), these advances may not translate to Autistic youth, leaving a critical gap in both research and clinical practice. Taken together, the systemic exclusion of Autistic youth underscores the urgent need for tailored research and interventions that address their unique risk factors and service needs.

### 4.2 Aim 2: identifying core intervention strategies for autistic youth

Several strategies were common across interventions and should be examined in future research to investigate their applicability to Autistic youth, as well as relevant adaptations.

#### 4.2.1 Follow-up contact

The most frequently used strategy was follow-up contact with the patient post-discharge. Follow-up is considered an important part of providing healthcare to families of Autistic youth, as it provides individualized support, monitors a youth’s health and treatment progress, and minimizes barriers to healthcare access (e.g., facilitates care navigation; 92). Repetition can also play a crucial role in learning for youth with executive functioning difficulties (93), and follow-up contact post-discharge may act as an opportunity to reinforce coping skills and concepts previously learned. In youth populations, follow-up that actively involves caregivers may be especially important, as family engagement can support adherence to recommendations, facilitate skill generalization at home, and enhance overall treatment effectiveness (94–96). Nevertheless, certain considerations may need to be made to follow-up contact protocols to be optimal for Autistic youth. For instance, protocols should emphasize consistency, predictability, and flexibility in communication. Autistic people may prefer face-to-face or written modes over phone calls (97), suggesting email, text, or other preferred formats may improve accessibility.

#### 4.2.2 Safety planning

Over half of identified interventions used safety planning. Safety planning may be well-suited to Autistic youth given its structured nature (27), and an autism-adapted safety plan has been developed for Autistic adults (28). Suggested adaptations for Autistic youth include integrating circumscribed interests, visual aids, and concrete instructions, while being sensitive to autism-related challenges like sensory overload and autistic burnout (52). Certain examples of coping strategies may also be tailored to be more relevant to Autistic youth (e.g., engaging with online communities rather than calling a friend; 27).

#### 4.2.3 Teaching coping skills

Many interventions emphasized teaching youth skills to help them cope with painful emotions. Autism-adapted mental health interventions often already use similar strategies, particularly cognitive behavioural therapy (CBT)-based interventions (e.g., 48, 98–101). For instance, Facing Your Fears, a CBT-based group for Autistic youth reduces youth anxiety through relaxation, emotion regulation, and graded exposure, with certain modifications to meet the cognitive, communication, and social needs of Autistic youth (48).

A recent systematic review (99) found that common autism-specific adaptations to interventions included emphasizing parent involvement (as appropriate), using visuals, incorporating circumscribed interests, using reinforcers, following structured formats and schedules, using more concrete and simplified language, and tailoring to the functional level of the individual. Overall, the teaching of emotional coping skills is an important component in several existing evidence-based and autism-adapted mental health interventions. Thus, similar strategies and adaptations may be transferrable to an autism-adapted brief suicide intervention.

#### 4.2.4 Providing psychoeducation

Some interventions included informational sessions or materials providing education about suicidal thoughts and behaviours, risk factors, and the seriousness of suicide. For Autistic youth, psychoeducation should address the unique presentation of suicidality among Autistic people. A toolkit based on research findings, perspectives from Autistic advocates, and expert consensus (54) has recently been developed to help identify signs of imminent suicidal behaviour among Autistic individuals. For example, while many Autistic people value time alone, sudden or increased social withdrawal may signal suicide risk (23). Autistic people are also more likely to experience traumatic events in their lifetime than non-Autistic people and perceive different kinds of events as traumatic (e.g., social events, changes in routine or environment; 102). This information is extremely valuable for family members and others close to an Autistic person to identify when they may be at high suicide risk, and when intervention may be warranted. Additionally, Autistic youth may be more likely to ruminate and perseverate on thoughts of suicide. These “sticky” thoughts may last for long periods, and it may be very difficult for Autistic people to switch from these thoughts and images (54). In these cases, it may be helpful for family members to intervene and distract the individual from these distressing thoughts (e.g., engaging in preferred or shared activities; 63). Autistic youth may also struggle more with executive functioning challenges including impulsivity (50), as well as difficulty recognizing when they are becoming dysregulated and verging on emotional crisis (28), which may make education about lethal means restriction particularly important. Autism-specific psychoeducation is crucial for families to effectively support their Autistic youth, as well as to empower Autistic individuals with self-knowledge, reduce internalized stigma about suicidal thoughts and behaviours, and enable them to actively participate in treatment (102).

#### 4.2.5 Identifying and reinforcing protective factors

Several interventions emphasized identifying and highlighting youth and family strengths and protective factors. Research shows that more frequent use of strengths strongly predicts better quality of life and well-being among Autistic people; however, Autistic people often report lower awareness, use of their strengths, and self-esteem compared to non-Autistic peers (103, 104). Pelton and Cassidy (105) also found that adults with higher levels of autistic traits were more likely to experience thwarted belongingness (a painful sense of an unmet need of connection; 106, 107) and perceived burdensomeness, thereby increasing their risk of attempting suicide. Conversely, stronger solidarity and connection with the autism community is associated with better psychological well-being (108). Hence, strengths-based approaches and fostering belonging may be an important avenue for suicide prevention among Autistic people.

#### 4.2.6 Assessment

The brief interventions in our study often used tools and frameworks to assess suicide risk, as well as conceptualize underlying problems. However, characteristics common to autism, such as communication challenges, difficulties identifying and expressing emotions (alexithymia), and difficulty understanding the finality of death, make accurate detection of suicidality among Autistic people extremely challenging (109). Thus, clinicians feel significantly less confident screening for suicide risk in Autistic people compared to their non-Autistic peers (110). Moreover, suicide risk assessments designed for the general population may be interpreted differently by Autistic people and often show poor reliability and validity in this group (111, 112). In response, adapted tools have been developed, including the Social Behaviours Questionnaire – Autism Spectrum Conditions (SBQ-ASC; 90), and the SIDAS-Modified (SIDAS-M; 113), both created with input from Autistic adults and showing promising psychometric properties.

While these advances represent important progress for Autistic adults, there remains an urgent need for validated suicide risk assessments for Autistic children and adolescents, where no accurate tools currently exist (89, 114). Developing and implementing autism-specific assessments will be critical to accurately identify suicide risk and monitor changes over time in this population.

#### 4.2.7 Connecting to follow-up treatment

Several interventions connected youth to ongoing care post-discharge. Given the multitude of barriers that families of Autistic youth often face when accessing mental health care (16), facilitating linkages to long-term care in the community is extremely valuable. Unfortunately, many providers feel unequipped to treat Autistic patients due to a lack of training and resources (10).

Given the distinct triggers, warning signs, and presentations of suicidality in Autistic people, as well as the need for autism-adapted approaches to treatment, it is essential to refer Autistic youth at risk for suicide to healthcare providers that are willing and able to support them. As there may be an insufficient number of healthcare providers with such competencies, training and awareness initiatives about autism and co-occurring mental health issues may be necessary to ensure that Autistic patients receive high quality care. For example, a recent systematic review of specialized autism training programs for physicians (115) highlighted that such programs are associated with positive changes in physician knowledge and self-efficacy related to the care of Autistic patients.

### 4.3 Aim 3: summarizing evidence of intervention effectiveness

Evidence of brief intervention effectiveness was mixed, with only eight of the 22 studies measuring suicide-related outcomes (36%) indicating that participation in the intervention was linked to a reduction in suicide attempts or deaths, suicidal thoughts and behaviours, and/or rehospitalizations for mental health reasons. Of these studies, three were randomized controlled trials (RCTs) that were assessed to be of high quality (65; [BIC] 66 [ASAP+BRITE]; 69 [BIC]). These findings mirror those of Dobias et al. (36) who found that fewer than one-third of brief suicide interventions improved at least one suicide outcome.

While evidence supporting the effectiveness of brief interventions is currently limited, this is unfortunately not unlike other therapeutic alternatives such as CBT and dialectical behaviour therapy (DBT). In a meta-analysis of studies reflecting the last 50 years of interventions for suicide and non-suicidal self-injury (NSSI; 38), overall intervention effects were small across suicide outcomes, and despite a large increase in the number of RCTs conducted, intervention efficacy has not improved over the past several decades. While potentially similar in effectiveness, brief interventions have advantages over traditional full-length interventions in that they are less resource intensive, can support youth in a critical period of high risk, and can be delivered in a variety of contexts (36). Further, findings from the current review show that brief interventions were consistently feasible, acceptable, and able to both effectively link youth to follow-up care and reduce youth’s usage of emergency department (ED) and inpatient care.

### 4.4 Limitations and future directions

This review identified brief interventions used in the general youth population that met specified criteria, focusing on youth who had attempted suicide and presented to an ED or other healthcare setting. As such, it did not capture other important approaches that are potentially relevant to Autistic youth, including universal awareness and education initiatives, school-based interventions, and caregiver or gatekeeper training. Well-rounded supports tailored to Autistic youth experiencing the full spectrum of suicidality (e.g., ideation, plans, preparatory behaviour, attempts) or exhibiting risk factors for suicidality (e.g., NSSI, trauma, mental health conditions) will play an important role in suicide prevention and warrant further attention and investigation. Additionally, this review focused on youth between the ages of 15 and 24 years, though research shows that Autistic children as young as seven years experience suicidality (34). Future work should therefore prioritize the development and validation of interventions for children and younger adolescents.

Mixed findings and limiting supporting evidence underscore the need for further evaluation of brief interventions, for both Autistic and non-Autistic youth. The heterogeneity and use of multiple modalities make it challenging to discern what characteristics of an intervention (e.g., frequency, dose, format, content) may be most effective. Large-scale RCTs by independent research teams and direct comparisons of intervention content and modalities will help clarify what approaches should be prioritized. Additionally, the temporal focus of the included studies varied, with some interventions targeting youth in the immediate post-attempt period and others recruiting participants based on lifetime attempt history or acute suicide risk. This heterogeneity reflects limitations in the existing literature and underscores the need for clearer timelines and definitions in future research on post-discharge suicide prevention.

Finally, community-based participatory research offers a powerful framework for advancing suicide prevention efforts among Autistic youth. By actively partnering with Autistic individuals, caregivers, and community members throughout all stages of the research process, from question development to intervention design and implementation, participatory methods ensure research is aligned with the lived experiences, priorities, and values of the community (116). Such approaches can improve engagement, intervention acceptability, and feasibility while addressing historical exclusion from mainstream mental health research that has contributed to systemic disparities (117). Integrating participatory research principles into suicide prevention research for Autistic youth could not only enhance study quality but promote co-ownership of interventions, reduce barriers to care, and ultimately improve outcomes for this historically underserved population (33, 83, 118).

## 5 Conclusion

The high prevalence of suicide attempts and deaths among Autistic youth, coupled with the distinct presentation of suicidality among these individuals, necessitates the development of autism-specific suicide intervention strategies. The current systematic review identified several brief interventions used in non-Autistic youth post-discharge from acute care for a suicide attempt. Information about the effectiveness of these interventions, as well as the quality of the evidence presented, provides insight into what interventions and strategies may be the most promising candidates for adaptation for use with Autistic youth. The information from this review, as well as guidance from Autistic youth and other community members, will be key in future development of autism-adapted brief suicide interventions.

## Data Availability

The original contributions presented in the study are included in the article/supplementary material, further inquiries can be directed to the corresponding author/s.

## Footnotes

1 Currently, there is no single, universally accepted way of describing autism (2); however, there is mounting evidence that most Autistic people prefer identity-first language (3, 4). Consequently, identity-first language is used throughout this manuscript to acknowledge the preferences of the majority of the Autistic community.
